# (-)-Gallocatechin Gallate Mitigates Metabolic Syndrome-Associated Diabetic Nephropathy in *db/db* Mice

**DOI:** 10.3390/foods13111755

**Published:** 2024-06-03

**Authors:** Xin Xiao, Huifang Ge, Yijun Wang, Xiaochun Wan, Daxiang Li, Zhongwen Xie

**Affiliations:** 1 State Key Laboratory of Tea Plant Biology and Utilization, Anhui Agricultural University, 130 Changjiang West Road, Hefei 230036, China; 2021150@ahau.edu.cn (X.X.); ghfjulia@ahau.edu.cn (H.G.); yijun@ahau.edu.cn (Y.W.); xcwan@ahau.edu.cn (X.W.); dxli@ahau.edu.cn (D.L.); 2 Joint Research Center for Food Nutrition and Health of IHM, Hefei 230036, China

**Keywords:** metabolic syndrome, diabetic nephropathy, GCG, renal transcriptome analysis

## Abstract

Metabolic syndrome (MetS) significantly predisposes individuals to diabetes and is a prognostic factor for the progression of diabetic nephropathy (DN). This study aimed to evaluate the efficacy of (-)–gallocatechin gallate (GCG) in alleviating signs of MetS-associated DN in *db/db* mice. We administered GCG and monitored its effects on several metabolic parameters, including food and water intake, urinary output, blood glucose levels, glucose and insulin homeostasis, lipid profiles, blood pressure, and renal function biomarkers. The main findings indicated that GCG intervention led to marked improvements in these metabolic indicators and renal function, signifying its potential in managing MetS and DN. Furthermore, transcriptome analysis revealed substantial modifications in gene expression, notably the downregulation of pro-inflammatory genes such as *S100a8*, *S100a9*, *Cd44*, *Socs3*, *Mmp3*, *Mmp9*, *Nlrp3*, *IL*–*1β*, *Osm*, *Ptgs2*, and *Lcn2* and the upregulation of the anti-oxidative gene *Gstm3*. These genetic alterations suggest significant effects on pathways related to inflammation and oxidative stress. In conclusion, GCG demonstrates therapeutic efficacy for MetS–associated DN, mitigating metabolic disturbances and enhancing renal health by modulating inflammatory and oxidative responses.

## 1. Introduction

Metabolic syndrome (MetS) encompasses a cluster of metabolic abnormalities triggered by visceral obesity and centered on insulin resistance, which induces abnormal metabolic components in MetS [1]. Recognized diagnostic criteria endorsed by international authorities identify central obesity, hypertension (≥130/85 mmHg), elevated fasting blood glucose (≥5.6 mmol/L), high triglyceride levels (≥1.7 mmol/L), and low HDL cholesterol as cardinal markers [2]. Epidemiologically, MetS impacts nearly 25% of the adult population globally [3], featuring adiposity, insulin resistance, hypertension, hyperglycemia, and dyslipidemia, which are not just risk factors for diabetes but also closely linked to its complications [4]. The rising incidence of diabetic nephropathy (DN) worldwide has drawn significant attention due to its status as a severe complication of diabetes [5]. DN not only affects kidney function but also increases the risk of cardiovascular disease and neuropathy, negatively impacting patient health. The pathological progression of DN involves complex biological mechanisms driven by persistent hyperglycemia, including glomerular matrix proliferation, capillary wall thickening, increased glomerular permeability, tubular epithelial cell injury, and interstitial fibrosis, which are exacerbated by oxidative stress and inflammation, culminating in renal damage [6].

Insulin resistance contributes to hyperglycemia, a key factor in DN pathogenesis [7,8], while hypertension induces detrimental changes in renal arterioles and glomeruli [8]. Although low HDL cholesterol and elevated triglycerides may not directly incite DN, they exacerbate vascular injury and inflammation, potentiating DN progression [9]. Furthermore, MetS is increasingly recognized as a dependent risk factor and prognostic marker for DN progression [10,11], highlighting the cruciality of addressing MetS to mitigate DN progression.

Tea–derived bioactive compounds, renowned for their antioxidative and anti-inflammatory properties, are increasingly utilized in DN intervention and treatment alongside synthetic drugs [12]. Epigallocatechin gallate (EGCG), derived from green tea, possesses antioxidative and anti–inflammatory properties, reducing body mass index and waist circumference and improving lipid profiles and cardiovascular health markers. These benefits, validated in preclinical and clinical studies, highlight the promising potential of EGCG against metabolic syndrome [13]. Nonetheless, the constrained bioavailability of EGCG has limited its clinical use [14]. (-)–Gallocatechin gallate ((−)-GCG), a stable EGCG stereoisomer, is noted for its superior efficacy in ameliorating hyperglycemia and antioxidative effects [15]. Despite prior investigations indicating the significant promise of GCG in ameliorating DN, surpassing EGCG [16], further research is needed to understand its potential and underlying mechanisms in modulating MetS to mitigate DN progression under diabetic conditions.

The *db/db* mouse model, characterized by diabetes and obesity due to the *db* gene mutation, induces insulin resistance and hyperglycemia [17]. It serves as an ideal platform for studying MetS–associated DN progression [18,19], which is particularly relevant for diabetic nephropathy patients manifesting these metabolic aberrations. Consequently, this study employs the *db/db* mouse model to explore the effects and mechanisms of (-)-gallocatechin gallate (GCG) in modulating metabolic syndrome to ameliorate DN, especially in the context of concurrent MetS, under diabetic conditions.

## 2. Materials and Methods

### 2.1. Animal Experiments

Sixteen male C57BLKsJ-*db*/+ (WT) and sixteen male C57BLKsJ-*db/db* (*db/db*) mice, all aged six weeks, were procured from the experimental animal supplier Changzhou Cavens Laboratory Animal Co., Ltd. These mice were accommodated in groups of4 per cage (8 animals/group) under specific pathogen–free (SPF) laboratory conditions at Anhui Agricultural University, where the environment was maintained at 23 °C ± 2 °C with a humidity level of 50% ± 5% and subjected to a 12:12 h light/dark cycle, with lights turned on from 8:00 a.m. to 8:00 p.m. They were provided with standard chow obtained from Trophic Animal Feed, Nantong, China, and had unrestricted access to water.

GCG (CAS#4233-96-9, ≥90% purity) was purchased from Chengdu Herbpurify Co., Ltd. (Chengdu, Sichuan, China). Following a 2–week acclimatization period, the mice were divided into four groups: WT control (WT, *n* = 8), WT + GCG intervention (WT + GCG, *n* = 8), *db/db* control (*db/db*, *n* = 8), and *db/db* + GCG intervention (*db/db* + GCG, *n* = 8). The mice underwent a 20–week treatment regimen, with the GCG–treated groups receiving an oral gavage of 100 mg/kg/day of GCG dissolved in distilled water by vortexing, while the control mice were administered an equivalent amount of water. During the intervention, the food consumption and water intake of each group of mice were monitored daily, while their body weight was measured weekly.

### 2.2. Body Composition Measurements

A Bruker LF90 II Minispec TD–NMR system (Bruker Optics, Billerica, MA, USA) was utilized for body composition analysis. The system operated with a magnetic field strength of 0.15 T and a pulse frequency of 6.2 MHz, providing data on percentages of body fat (% fat) and lean tissue (% lean), fat mass, and lean mass.

### 2.3. Glucose Tolerance Test and Insulin Resistance Test

Glucose tolerance tests (GTT) and insulin resistance tests (IRT) were conducted after 14 and 16 weeks of intervention, respectively. Briefly, mice underwent a 6 h fasting period, followed by intraperitoneal injections of glucose (1.5 g/kg body weight) or insulin (1 U/kg body weight). Tail vein blood glucose levels were assessed at 0, 30, 60, 90, and 120 min intervals using the Alpha TRAK2 Blood Glucose Monitoring Kit (Zoetis, Parsippany, NJ, USA). In the prediabetic phase in humans, the fasting plasma glucose ranges between 100-125 mg/dL. In the diabetic phase, the fasting plasma glucose is 126 mg/dL or higher.

### 2.4. Lipid Profile Measurements

Blood samples were collected from mice following an overnight fasting period through a tail vein puncture. Plasma triglyceride (TG), low–density lipoprotein cholesterol (LDL–C), and total cholesterol (T–CHO) levels were measured using microtest kits sourced from Nanjing Jiancheng Bioengineering Institute (Nanjing, China).

### 2.5. Blood Pressure Measurement

Blood pressure in mice aged 26 weeks was noninvasively monitored via a tail cuff system (BP-2000) from Visitech Systems, Inc. (Apex, NC, USA), subsequent to a 5– to 7–day acclimatization and stabilization. Measurements were obtained from awake mice on a heated platform, with an average of 20 consecutive measurements used to calculate systolic blood pressure (SBP) and diastolic blood pressure (DBP) [20].

### 2.6. Determination of Renal Functions

The mice, aged 23 weeks, were housed in metabolic chambers (Nalgene) for 24 h durations, during which they had unrestricted access to food and water. Urine samples were collected at 12 h intervals at ZT4 and ZT16 (ZT (zeitgeber time) is a chronobiological instrument employed to harmonize intrinsic biological rhythms with external light/dark oscillations). The levels of urinary microalbumin (mALB), kidney injury molecule−1 (KIM−1), and neutrophil gelatinase-associated lipocalin (NGAL) were measured using ELISA kits from Shanghai Jianglai Biotechnology Co., Ltd., Shanghai, China. The animal facility had a 12:12 h light/dark cycle (lights on from 8:00 a.m. to 8:00 p.m.), with ZT4 at 12:00 p.m. and ZT16 at 12:00 a.m.

### 2.7. Histological Examination

Kidney tissues were preserved in 10% neutral buffered formalin before being embedded in paraffin and then sectioned into 5 μm thick slices using a fully automated rotary microtome, specifically the LEICA RM2255. Slides were stained with hematoxylin and eosin using a kit from Beijing Solarbio Sciences & Technology Co., Ltd., Beijing, China. Imaging was conducted with an Olympus VS2000 slide scanner and analyzed via Image−J 2 software (NIH, Bethesda, MD, USA).

### 2.8. Transcriptome Analysis of Renal Tissues

Kidney samples (*n* = 4 each) from the WT, WT + GCG, *db/db*, and *db/db* + GCG groups, following a 20−week intervention, underwent total RNA extraction with TRIzol^®^ reagent (Invitrogen, Carlsbad, CA, USA). Subsequently, RNA was subjected to sequencing using the Illumina HiSeq X Ten platform. Differential expression analysis was carried out to identify genes with significant changes (|log2FC| ≥ 1, *p* < 0.05) employing DESeq2. Gene Ontology (GO) and Kyoto Encyclopedia of Genes and Genomes (KEGG) enrichment analyses, corrected using the Bonferroni method with a significance threshold of *p* ≤ 0.05, were conducted utilizing GOATOOLS and KOBAS [21]. The analysis of protein–protein interactions (PPIs) among differentially expressed genes (DEGs) was conducted using the STRING database (https://cn.string-db.org/) accessed on 27 December 2023. The overall data analysis (excluding PPIs) was executed on the Majorbio Cloud platform (https://cloud.majorbio.com) accessed on 27 December 2023.

### 2.9. Statistical Analysis

Statistical analyses included two–group comparisons using a 2–tailed Student’s *t*–test and multiple group comparisons using a one–way ANOVA followed by Tukey’s test. A significance threshold of *p* < 0.05 was applied. Data are presented as mean ± SEM.

## 3. Results

### 3.1. GCG Alleviating Type 2 Diabetes Sign in db/db Mice

The *db/db* mouse model is widely employed for investigating type 2 diabetes (T2D), which is characterized by hallmark signs including hyperphagia, polydipsia, polyuria, and subsequent weight loss [22].

**Figure 1:**
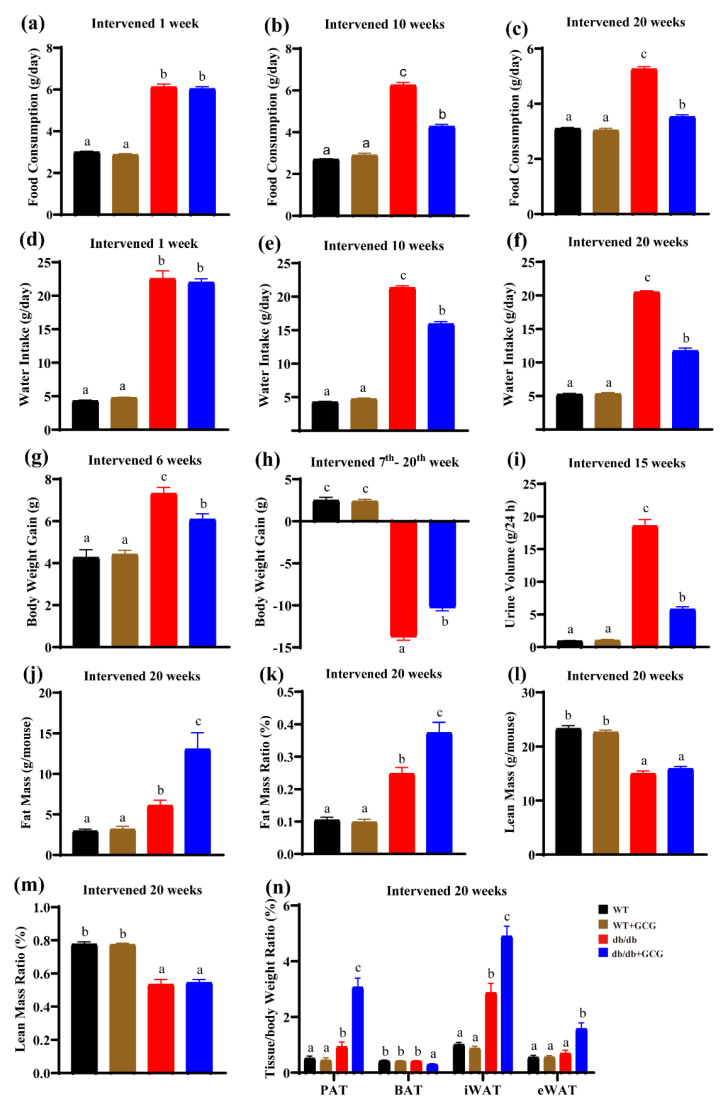
Measurement of food consumption, water intake, 24 h urinary volume, body weight gain, body composition, and tissue/body weight ratio in mice from all groups. (**a**–**c**) food consumption after intervention for 1, 10, and 20 weeks; (**d**–**f**) water intake after intervention for 1, 10, and 20 weeks; (**g**) body weight gain following intervention for 6 weeks; (**h**) body weight gain from 7th to 20th week intervention; (**i**) 24 h urine volume after intervention for 15 weeks; (**j**,**k**) fat mass and fat mass ratio; (**l**,**m**) lean mass and lean mass ratio; (**n**) tissue/body weight ratio. PAT: perirenal adipose tissue; BAT: brown adipose tissue; iWAT: inguinal white adipose tissue; eWAT: epididymal white adipose tissue. WT: normal control; *db/db*: diabetic model control; Values are presented as means ± SEM (*n* = 8). Different letters above the bars denote significant differences (*p* < 0.05) between groups.

In our study, we monitored daily food consumption and water intake, which were significantly elevated in *db/db* mice compared to their WT counterparts. Notably, the administration of GCG resulted in a substantial reduction in both food consumption and water intake among *db/db* mice (Figure 1a–f). Additionally, following 15 weeks of GCG intervention, a significant reduction in the elevated 24 h urine volume was observed in *db/db* mice in comparison to the WT control group (Figure 1i). These results indicate the potential involvement of GCG in improving T2D complications.

Furthermore, we performed weekly measurements of body weight, revealing a gradual increase in body weight in *db/db* mice during the early stage of diabetes, spanning from 8 to 14 weeks of age (intervened for 6 weeks) (Figure 1g). Subsequently, these mice exhibited persistent weight loss in the late stage of diabetes, occurring between 15 and 28 weeks of age (intervened from the 7th to the 20th week) (Figure 1h), which is indicative of the development of diabetic complications. Remarkably, GCG treatment effectively countered this trend, mitigating weight gain in both the early and late stages of diabetes. These findings suggest that GCG has the capacity to prevent excessive weight gain in *db/db* mice.

The proportion of lean tissue and body fat ratio indicate the relative distribution of lean tissue to total body weight within each mouse group. Higher lean tissue proportions typically suggest lower body fat percentages, reflecting better overall health. This metric is commonly used to assess the health and nutrition of animal models in research [23]. Our analysis of body composition after 20 weeks of intervention indicated no significant differences in lean mass content or lean mass ratio between the *db/db* model group and the GCG-intervened *db/db* group of mice. However, GCG intervention in *db/db* mice significantly prevented the decline in fat content and fat percentage (Figure 1j,k) in the late stage of diabetes.

In mice, adipose tissue comprises white adipose tissue (WAT), including iWAT and eWAT, which stores energy, and brown adipose tissue (BAT), which promotes energy expenditure [24]. Additionally, perirenal adipose tissue (PAT), surrounding the kidneys, is involved in renal function and metabolic regulation [25]. Figure 1n provides insights into the tissue/body weight ratios, demonstrating that the PAT, iWAT, and eWAT of GCG-intervened *db/db* mice exhibited significantly higher values compared to those of the *db/db* model group mice. Conversely, the BAT of *db/db* model group mice showed significantly higher values than those of the GCG–intervened *db/db* group mice. These results underscore that GCG treatment contributes to the prevention of weight loss in the later stages of diabetes by preserving adiposity (PAT, iWAT, and eWAT) in *db/db* mice.

### 3.2. GCG Prevents Metabolic Syndrome in db/db Mice

**Figure 2:**
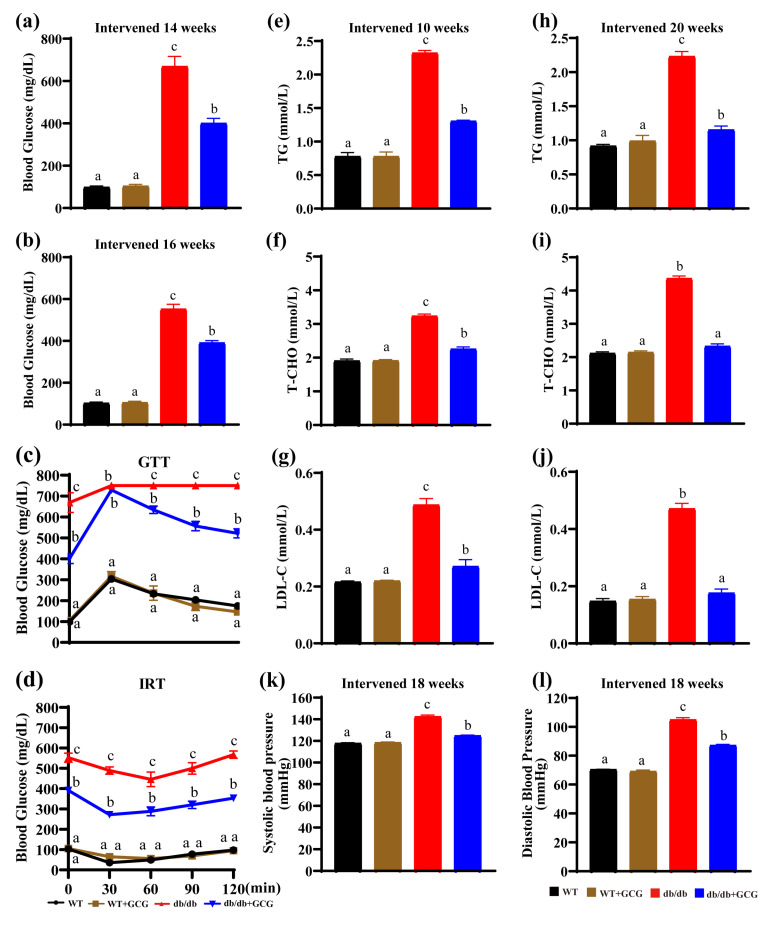
GCG prevents risk factors for MetS in *db/db* mice. (**a**,**b**) Fasting blood glucose levels after intervention for 14 and 16 weeks, respectively; (**c**) GTT levels and (**d**) IRT levels after intervention for 14 and 16 weeks, respectively; (**e**,**h**) TG after intervention for 10 and 20 weeks; (**f**,**i**) T–CHO after intervention for 10 and 20 weeks; (**g**,**j**) LDL–C after intervention for 10 and 20 weeks; (**k**) SBP; (**l**) DBP. WT: normal control; *db/db*: diabetic model control. Values are presented as means ± SEM (*n* = 4–5). Different letters above the bars denote significant differences (*p* < 0.05) between groups.

The key markers of MetS, including hyperglycemia, hypertension, high plasma triglyceride levels, and low HDL cholesterol, are recognized modifiable risk factors linked to DN progression [8]. Thus, we investigated these metabolic aberrations. Fasting blood glucose levels in *db/db* mice continuously demonstrated an increase in comparison to the WT group throughout the entire experimental period. However, treatment with GCG resulted in significant reductions in blood glucose levels, as evidenced by substantial decreases observed after 14 and 16 weeks of intervention in *db/db* mice (Figure 2a,b).

Furthermore, our findings from the GTT revealed markedly impaired glucose tolerance in *db/db* mice. In contrast, GCG treatment significantly ameliorated glucose tolerance in *db/db* mice, supported by a marked improvement in glucose tolerance (Figure 2c). As a reflection of insulin homeostasis, the IRT indicated impairment in *db/db* mice, manifesting as increased blood glucose levels following insulin injections. Importantly, GCG interventions induced significant reductions in blood glucose levels at each time point, demonstrating the effective improvement in insulin tolerance in *db/db* mice (Figure 2d).

Proceeding to the assessment of the lipid profile, Figure 2e–j illustrate that the plasma levels of TG, T–CHO, and LDL–C were notably higher in the *db/db* model group in comparison to the WT group. However, following 10 and 20 weeks of GCG intervention, these parameters exhibited significant reductions, underscoring the lipid-regulating potential of GCG.

Additionally, our observations revealed that *db/db* mice aged 26 weeks displayed a higher SBP of 142.47 ± 1.42 mmHg and a higher DBP of 105.0 ± 1.38 mmHg in comparison to WT mice, whose SBP measured 117.8 ± 0.62 mmHg and DBP measured 70.40 ± 0.22 mmHg, suggesting the existence of hypertension in *db/db* mice. Impressively, following an 18-week period of GCG intervention, significant reductions in both SBP (124.9 ± 0.54 mmHg) and DBP (DBP: 87.11 ± 0.69 mmHg) were observed in mice in the *db/db* + GCG group (Figure 2k,l), suggesting the potential of GCG in mitigating hypertension.

These results highlight the substantial benefits of GCG intervention in modulating hyperglycemia, improving glucose tolerance, regulating lipid profiles, and reducing hypertension in *db/db* mice. This signifies its potential therapeutic value in mitigating the progression risk factors of DN by addressing key MetS indicators within the context of T2D.

### 3.3. GCG Improves Urinary Renal Function Biomarkers in db/db Mice

**Figure 3:**
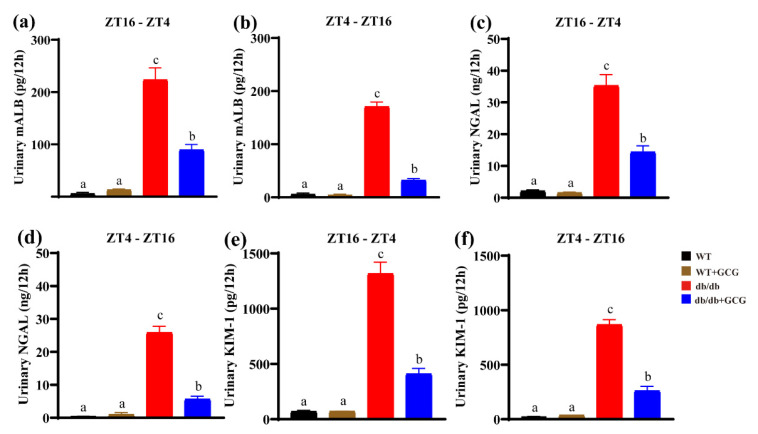
Levels of key renal function markers measured in mice from the experimental groups following a 16–week intervention at ZT16–ZT4 and ZT4–ZT16, respectively. (**a**,**b**) Urinary mALB level; (**c**,**d**) urinary NGAL level; (**e**,**f**) urinary KIM-1 mass. WT: normal control; *db/db*: diabetic model control. Values are presented as means ± SEM (*n* = 4–5). Different letters above the bars denote significant differences (*p* < 0.05) between groups.

The kidney functions as a pivotal biological clock organ, manifesting circadian rhythms in processes such as urine formation and excretion [26,27]. Based on prior research, *db/db* mouse kidneys exhibit circadian rhythms, with active and inactive phases [28]. Urine samples were collected at two 12 h intervals: ZT16−ZT4 and ZT4−ZT16. The levels of key renal function markers were assessed to evaluate the effects of GCG on renal function. In our study, the *db/db* mice displayed elevated urinary mALB (Figure 3a,b), NGAL (Figure 3c,d), and KIM−1 (Figure 3e,f) compared to WT at both intervals, signifying glomerular and tubular impairment. After 16 weeks of GCG treatment, these markers significantly decreased, suggesting the potential of GCG in mitigating renal injury during different circadian phases.

### 3.4. GCG Improved the Renal Structure in db/db Mice

**Figure 4:**
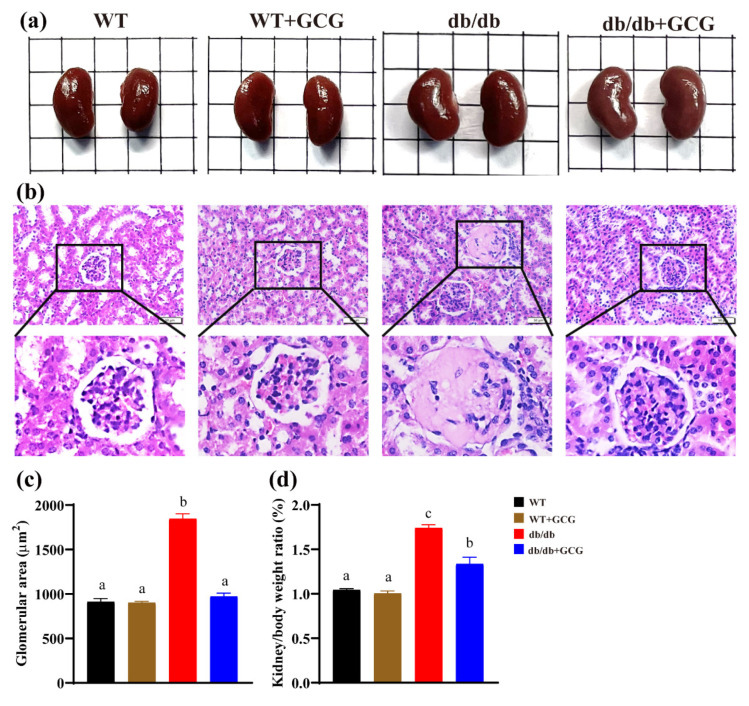
Analysis of renal structure in mice from each experimental group. (**a**) Representative kidney photos; (**b**) representative HE staining of glomeruli, magnified at 400× with a scale bar of 50 μm; (**c**) quantification of glomerular area in HE–stained kidney sections, with 30 glomerular sections in each group; (**d**) weight–to–body weight ratio. WT: normal control; *db/db*: diabetic model control. Values are presented as means ± SEM (*n* = 4–5). Different letters above the bars denote significant differences (*p* < 0.05) between groups.

MetS substantially amplifies susceptibility to DN, consequently expediting the genesis of glomeruli lesions. In our study, renal HE staining displayed that *db/db* mice had hyalinoid lesions of the mesangial matrix (Figure 4b) and significantly increased glomerular area (Figure 4c) when compared to the WT group. Additionally, the discernible elevation in renal hypertrophy, as indicated by the significantly higher kidney–to–body weight ratio in *db/db* mice than their WT counterparts (Figure 4d), correlated with these structural aberrations. However, the intervention of GCG for 20 weeks exhibited promising prospects in mitigating abnormal renal hypertrophy and enhancing glomerular structural integrity within the realm of diabetic kidney disease, thereby underscoring its potential as a therapeutic intervention.

### 3.5. Evaluation of Gene Expression and Identification of DEGs among Sample Sets

Prior to RNA sequencing, a comprehensive assessment of the total RNA extracted from the kidney was conducted. This assessment encompassed purity, concentration, integrity, and alignment to the genome (Table S1). Results consistently met the following criteria: OD 260/280 ratios were ≥2.01, OD 260/230 ratios were ≥2.25, concentration exceeded 1000 ng/μL, and RQN values were ≥8.7 for all samples. These assessments affirmed the suitability of RNA for sequencing. A total of 751,820,570 raw reads and 746,171,960 clean reads were generated, with a low error rate (0.026%) and high quality (Q20: 97.56%, Q30: 92.94%). GC content consistency within sample groups was noted (Table S2). Clean reads were aligned with the Mus_musculus reference genome, with total mapping >96%, multiple mapping 8.3–10.9%, and uniquely mapped reads 85.92–88.62% (Table S3). Our RNA sequencing quality control ensures data reliability and appropriate reference genome usage.

**Figure 5:**
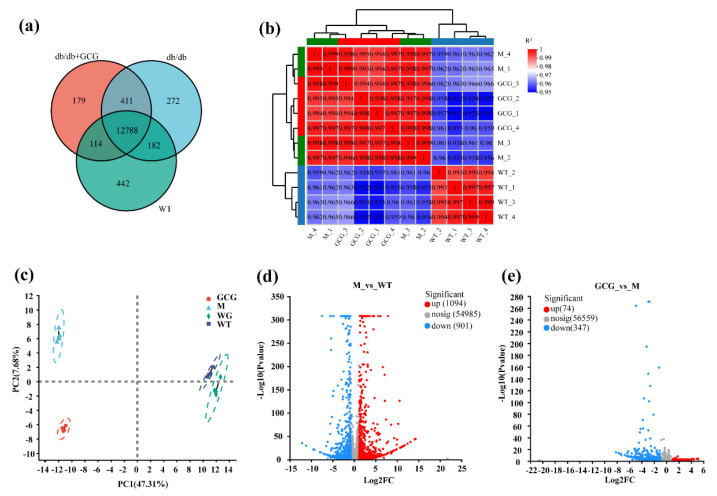
Assessment of gene expression and identification of DEGs among renal samples from the experimental groups. (**a**) Comparison analysis among various experimental groups using Venn diagrams; (**b**) examination of sample correlation from various experimental groups; (**c**) PCA conducted on samples derived from diverse experimental groups; (**d**) volcano plot of comparison of gene expression between M and WT groups (M as the control); (**e**) comparison of gene expression between GCG and M groups. Upregulated genes are denoted by red dots, downregulated genes by green dots, and non-significantly different genes by gray dots. Kidney samples from WT (WT_1–WT_4), WT + GCG (WG_1–WG_4), *db/db* (M_1–M_4), and *db/db* + GCG (GCG_1–GCG_4) groups are included; PCA, principal component analysis.

The gene expression analysis consisted of Venn analysis, correlation analysis, and principal component analysis (PCA). In the Venn analysis (Figure 5a), we identified 12,788 genes that were common to the M, WT, and GCG groups, with 272, 442, and 179 unique genes in the respective groups. Subsequently, in the correlation analysis (Figure 5b), all groups exhibited robust correlation coefficients exceeding 0.9, indicative of a high degree of concordance in gene expression across different experimental conditions. The PCA analysis (Figure 5c) provided insights into sample variations and the relative contributions of principal components. Principal component 1, contributing 47.31% of the variability, emerged as a major driver, while principal component 2 contributed 7.68%. The graphical representation effectively portrayed distinct separations among the various groups, notably emphasizing the substantial disparity between the GCG and M groups. This underscores the profound impact of GCG intervention on mRNA expression profiles. Figure 5d unveiled that under conditions of diabetes induction, a total of 1995 genes exhibited significant differential expression, encompassing 1094 upregulated and 901 downregulated genes. In the GCG intervention group, 421 differentially expressed genes (DEGs) were identified compared to the M group, with 74 being upregulated and 347 being downregulated (Figure 5e). Collectively, these rigorous analyses contribute significantly to our comprehension of the influence of both diabetes induction and GCG intervention on gene expression profiles.

### 3.6. Gene Set Functional Annotation and Enrichment Analysis

**Figure 6:**
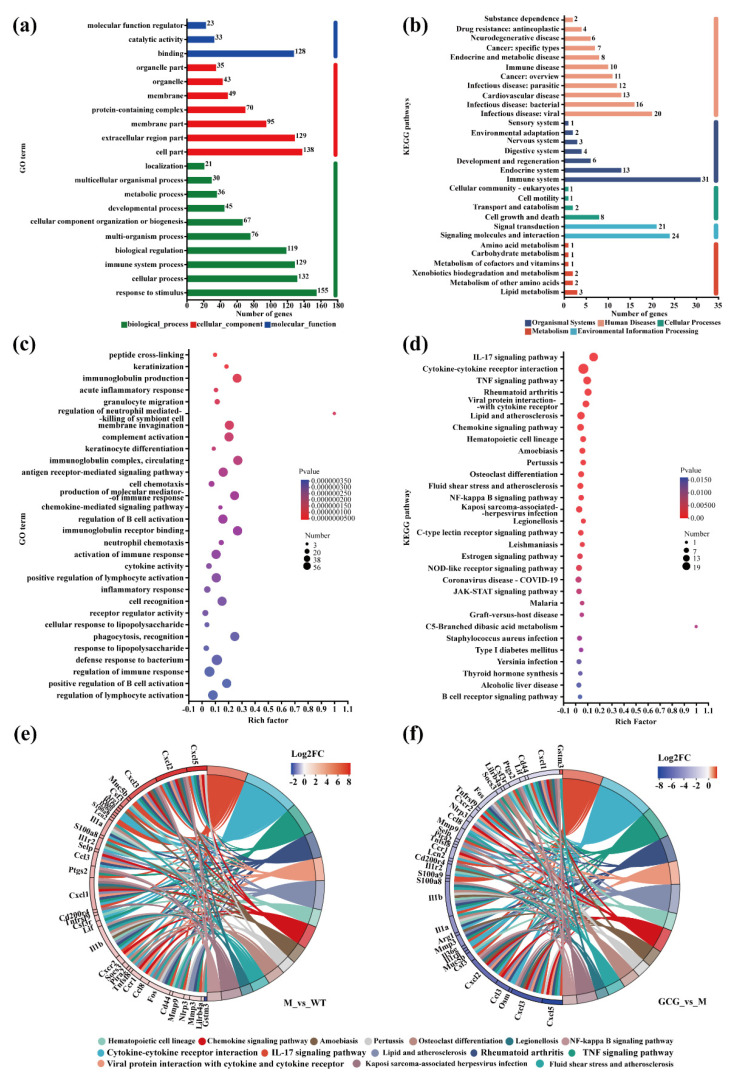
Functional annotation and enrichment assessment of gene sets within the experimental groups. (**a**) GO annotation of gene set; (**b**) KEGG pathway annotation of gene set; (**c**) GO enrichment of gene set; (**d**) enrichment analysis of gene sets within KEGG pathways; (**e**,**f**) chord diagram illustrating enriched KEGG pathway interactions between M and WT groups (with WT as the control) and between M and GCG groups (with M as the control), respectively.

Our investigation commenced by identifying DEGs with distinct patterns in diabetes-induced and intervention groups. These DEGs were categorized into a gene set (Table S4) and subjected to Gene Ontology (GO) analysis, revealing their involvement in diverse stimuli-responsive biological processes, predominantly localized within cellular components such as cell parts, and primarily associated with molecular functions involving various binding activities (Figure 6a).

Subsequently, Kyoto Encyclopedia of Genes and Genomes (KEGG) pathway analysis classified these DEGs into six major domains, prominently highlighting the immune system, signaling molecules, interactions, and viral infectious disease pathways. These pathways are intricately correlated with immune responses, cellular signaling, and mechanisms underpinning infectious disease (Figure 6b).

GO enrichment analysis further provided further insights into the core functionalities of these genes, underscoring their active participation in inflammation and oxidative stress, such as acute inflammatory responses and reactions to lipopolysaccharides (Figure 6c).

Furthermore, KEGG functional enrichment analysis accentuated the pivotal relevance of these genes within pathways intricately linked to inflammation and oxidative stress, TNF signaling, JAK–STAT signaling, IL–17 signaling, and cytokine–cytokine receptor interactions (Figure 6d).

In summary, our analysis reveals the biological significance of these DEGs, particularly within the critical functional processes and pathways associated with inflammation and oxidative stress in the context of diabetes and intervention.

### 3.7. Mechanisms of GCG in Preventing Diabetic Kidney Injury

Based on the findings of the transcriptome analysis, we observed alterations in gene expression associated with diabetic kidney injury, impacting multiple pathways and leading to abnormal protein expression. To delve deeper into these findings, we generated an enriched chord diagram illustrating the top 15 KEGG pathways (Figure 6e). After intervention with GCG (Figure 6f), with the M group as the control, only Gstm3 was significantly upregulated, while *S100a8*, *S100a9*, *Fos*, *Socs3*, *Il1β*, etc. were significantly downregulated. Notably, the majority of these genes exhibited potential for mitigating kidney damage induced by diabetes through the regulation of inflammation- and oxidation-related genes.

The chemokine (C–C motif) ligand 3 (Ccl3), chemokine (C–C motif) ligand 8 (Ccl8), chemokine (C–C motif) receptor 1 (Ccr1), colony-stimulating factor 3 (granulocyte) (Csf3), colony–stimulating factor 3 receptor (granulocyte) (Csf3r, chemokine (C–X–C motif) ligand 1 (Cxcl1), chemokine (C–X–C motif) ligand 2 (Cxcl2), chemokine (C–X–C motif) ligand 3 (Cxcl3), chemokine (C–X–C motif) ligand 5 (Cxcl5), chemokine (C–X–C motif) receptor 2(Cxcr2), FBJ osteosarcoma oncogene (Fos), interleukin 1 alpha (Il1a), interleukin 1 beta (Il1b), interleukin 1 receptor type II (Il1r2), interleukin 1 receptor antagonist (Il1rn), interleukin 36G (Il36g), lipocalin 2 (Lcn2), matrix metallopeptidase 3 (Mmp3), matrix metallopeptidase 9 (Mmp9), mucin 5 subtype B, tracheobronchial (Muc5b), NLR family pyrin domain–containing 3 (Nlrp3), oncostatin M (Osm), paired Ig–like receptor A2 (Pira2), prostaglandin–endoperoxide synthase 2 (Ptgs2), S100 calcium-binding protein A8 (calgranulin A) (S100a8), S100 calcium–binding protein A9 (calgranulin B) (S100a9), suppressor of cytokine signaling 3 (Socs3), tumor necrosis factor receptor superfamily member 9 (Tnfrsf9), tumor necrosis factor (ligand) superfamily member 8 (Tnfsf8), leukemia inhibitory factor (Lif), and leukocyte immunoglobulin–like receptor subfamily B member 4A (Lilrb4a) were all involved in inflammation. The involvement of oxidation included the glutathione S-transferase, mu 3 (Gstm3), and lipocalin 2 (Lcn2).

## 4. Discussion

DN and MetS, linked by shared physiological mechanisms and risk factors like hypertension and hyperglycemia, may not always coexist in patients [10]. The *db/db* mouse model, with its genetic predisposition, serves as a classic model for studying the coexistence of MetS and DN [29]. In our study, the *db/db* mouse model exhibited hallmark features of T2D, including polydipsia, polyphagia, and polyuria (Figure 1). Furthermore, it progressed to a pathological state featuring concurrent MetS and DN, characterized by insulin resistance, hyperglycemia, hypertension, dyslipidemia (Figure 2), and renal structural abnormalities, including glomerular lesions and kidney hypertrophy (Figure 4).

As diabetic nephropathy progresses, *db/db* mice may manifest weight loss, which could be due to exacerbated insulin resistance and declining pancreatic β-cell function. These factors contribute to metabolic disturbances, increased energy expenditure, and depletion of muscle and adipose tissue [30]. GCG intervention improved insulin resistance (Figure 2) and enhanced energy utilization, possibly through direct or indirect effects on adipocyte function and distribution (Figure 1n), as well as adipose metabolism balance and even appetite regulation (Figure 1a–c), potentially leading to fat accumulation (Figure 1j,k). While the proportion of lean tissue did not significantly differ between the GCG-treated *db/db* group and the *db/db* model group (Figure 1m), the body fat percentage in the GCG−treated *db/db* group was notably higher (Figure 1k). Nevertheless, further experimental investigations are needed to elucidate these mechanisms.

MetS is experiencing a notable surge in worldwide prevalence, which is characterized by hyperglycemia due to insulin resistance, a key factor in diabetes and DN development [31]. In our study, we observed that GCG effectively lowered fasting blood glucose levels and improved both glucose and insulin homeostasis (Figure 2a–d) in *db/db* mice. This aligns with the findings of Xie et al., who observed comparable advantages of GCG concerning glucose tolerance and insulin sensitivity in a diabetic rat model induced by streptozotocin [32]. Therefore, the ameliorative effect of GCG on MetS and its associated progression towards DN may be attributed to its capacity to enhance insulin sensitivity, ultimately contributing to the regulation of glucose homeostasis. Moreover, the enhancement of insulin sensitivity by GCG may result from its inhibition of Socs3 and Ptgs2 gene expression; this hypothesis could be supported by our RNA−seq results (Figure 6).

MetS frequently encompasses aberrant lipid metabolism, characterized by elevated TG and reduced HDL–C levels. These lipid abnormalities are intricately associated with the onset and progression of DN. Notably, research has elucidated the indirect impact of fenofibrate, primarily tailored to reduce serum TG levels, in mitigating the severity of albuminuria among individuals with T2D, implying the contributory role of TG in the genesis and advancement of proteinuria [33]. In our study, we discerned a significant reduction in TG, T–CHO, and LDL–C levels in *db/db* mice following treatment with GCG (Figure 2e–j). The potential of GCG to alter TG levels is supported by a study conducted by Lee et al., which underscores the capacity of GCG to substantially lower TG and T-CHO levels in the hepatic tissue of rats subjected to a hyperlipidemic diet [34]. Additionally, research conducted by Xie et al. demonstrated the ability of GCG to reduce plasma TG levels induced by streptozotocin in rats [32]. Furthermore, earlier research has illustrated that GCG regulates the expression of genes implicated in TG synthesis, transport, and consumption in differentiated adipocytes and myocytes [35]. Thus, these findings indicate that GCG may improve lipid metabolism by modulating TG–related gene expression, thereby reducing TG levels and potentially ameliorating both MetS and DN. 

MetS presents concomitantly with hypertension, wherein elevated SBP emerges as an independent risk factor for the development of DN. This hypertensive condition can expedite renal glomerular injury by augmenting pressure within the afferent arterioles [36]. In our study, GCG exhibited a notable capacity to significantly reduce SBP in *db/db* mice with an observable amelioration in renal glomerular pathology (Figure 2k,l). A preceding study has reported the potential of EGCG to lower SBP in *db/db* mice, possibly owing to its anti-inflammatory effects on the vasculature [37]. Li et al. found that GCG attenuates NF–κB in 3T3–L1 cells, reducing LPS–induced IL–6 and MCP–1 production, thus mitigating adipocyte inflammation [38]. Furthermore, as a stereoisomer of EGCG, GCG even demonstrated various superior bioactivities compared to its counterpart. This indirectly supports the notion that GCG may also lower SBP through its anti-inflammatory effects on blood vessels.

Insulin resistance serves as a fundamental hallmark of MetS, and within the kidneys, a pivotal organ in metabolic processes, diverse cells within both the glomerular and renal tubular compartments exhibit a dependency on insulin signaling [39]. Consequently, MetS can precipitate pathological alterations within these renal domains, encompassing glomerular sclerosis, reduced glomerular filtration rates, and tubular inflammation. These changes, in turn, affect the excretion of critical renal functional markers in urine. In diabetic conditions, mALB serves as an indicator of glomerular filtration, NGAL signifies early DN by mirroring tubular injury and inflammation, and KIM−1 denotes tubulointerstitial damage and renal interstitial inflammation [40]. Hence, in our study, the notable decrease in 24 h urine volume (Figure 1i) and the levels of NGAL, KIM−1, and mALB (Figure 3) in the 12 h urine samples of *db/db* mice following GCG intervention may be linked to its ability to modulate insulin homeostasis, ultimately contributing to the amelioration of renal structure and function. Moreover, in diabetes, chronic inflammation may disrupt the inflammatory status of PAT, leading to functional alterations or atrophy. This can impact renal blood flow, metabolic status, and PAT content and function. Hence, maintaining PAT content in GCG−treated *db/db* mice may be attributed to its anti−inflammatory effects (Figure 6).

Chronic inflammation and oxidative stress indisputably assume crucial roles in the development of MetS and kidney diseases such as DN. To illuminate the mechanism by which GCG intervenes in inflammation, we used several significantly expressed genes (*S100a8*, *S100a9*, *Cd44*, *Socs3*, *Mmp3*, *Mmp9*, *Nlrp3*, *IL-1β*, *Osm*, and *Ptgs2*) as examples. Prior research has established that S100A9 and S100A8 possess pro−inflammatory properties [41]. Furthermore, their heterodimer, S100A8/S100A9, promotes renal interstitial fibrosis in DN, facilitating the process of epithelial–mesenchymal transition (EMT) and fibrosis [42]. Consequently, the downregulation of S100A8 and S100A9 expression holds promise for diminishing the release of pro−inflammatory mediators, thereby attenuating the inflammatory response and ultimately ameliorating renal interstitial fibrosis.

Studies have indicated that the enrichment of CD44 in the injured proximal tubules indicates renal epithelial cell injury [43], with its reduction contributing to a decrease in renal fibrosis [44]. Research has shown that SOCS3 serves a pivotal role in modulating inflammation and regulating energy and glucose balance, potentially leading to increased appetite and insulin and leptin resistance across various metabolic conditions [45,46]. Importantly, attenuating SOCS3 expression levels has been associated with improved sensitivity to leptin and insulin [47], thereby yielding beneficial metabolic outcomes. 

MMP−9 has been demonstrated to upregulate TGF−β expression, thereby fostering the pathogenesis of renal interstitial fibrosis [48]. Changes in Mmp9 and Mmp3 expression/activity are linked to cardiovascular diseases, including hypertension, lipid disorders, and atherosclerosis [49]. Moreover, GCG has been demonstrated to mitigate tumorigenesis by inhibiting MMP−9 secretion through NF−κB pathway regulation, resulting in reduced MMP-9 promoter activity and mRNA expression [50]. 

Hyperglycemia, hyperlipidemia, and hyperuricemia trigger the NLRP3 inflammasome, linking metabolic stress in DN to pro-inflammatory responses via IL–1β and IL–18 [51,52]. IL–1β is central in DN, inducing ICAM-1 expression, increasing vascular permeability, and promoting ECM synthesis in the glomerular mesangium [53]. OSM induces fibrosis markers and inflammation factors in renal fibroblasts [54]. It also inhibits N-cadherin in proximal tubular cells through ERK1/2 signaling, promoting tubular epithelial cell mesenchymal transformation [55]. PTGS2 significantly contributes to insulin resistance and, when overexpressed, disrupts renal blood flow and hemodynamics [56], leading to increased renin receptor expression and podocyte injury [57]. Inhibiting PTGS2 has the potential to delay the onset and progression of DN. 

The intervention of oxidation involves the Gstm3 and Lcn2 genes. GSTM3 actively regulates ROS in proximal tubular epithelial cells, actively participating in oxidative stress pathology by facilitating ROS conjugation with glutathione [58]. Additionally, upregulation of Gstm3 boosts the ability to resist oxidative stress [59]. Lcn2 autonomously governs its expression and serves as an early biomarker for kidney injury, positively correlating with inflammation, innate immunity, and oxidative stress [43,60]. Altogether, GCG–regulated genes, whether through direct or indirect mechanisms, contribute to its action, particularly in addressing inflammation and oxidative stress, with inflammation being the predominant aspect. 

Based on our transcriptome KEGG functional enrichment analysis, the IL–17 signaling pathway emerged as the foremost among the enriched gene sets, featuring key genes such as *Ptgs2*, *Mmp9*, *Mmp3*, *S100a8*, *S100a9*, *Lcn2*, and *Il*–*1β*. Their modulation within this pathway may influence MetS and DN via effects on insulin signaling, inflammation, renal interstitial fibrosis, and renal tissue damage. Therefore, further research targeting the IL−17 pathway may be crucial for elucidating the mechanisms underlying the effectiveness of GCG against MetS−associated DN.

## 5. Conclusions

This study assessed the therapeutic potential of GCG in addressing MetS and DN using *db/db* mice. Our results demonstrated that GCG effectively mitigated key components of MetS, including hyperglycemia, dyslipidemia, and hypertension. It also improved T2D symptoms such as hyperphagia, polydipsia, polyuria, glucose, and insulin homeostasis while simultaneously positively impacting renal structure and function. Renal transcriptome analysis revealed that GCG modulates genes linked to inflammation and oxidative stress, pathways critically involved in the progression of MetS and DN. Overall, GCG emerges as a promising natural therapeutic agent for managing MetS−associated DN, meriting additional research and potential development into clinical applications.

## Data Availability

The original contributions presented in the study are included in the article/Supplementary Materials, further inquiries can be directed to the corresponding author.

## Supplementary Materials

The following supporting information can be downloaded at: https://www.mdpi.com/article/10.3390/foods13111755/s1, Table S1: Quality assessment of total RNA in kidney tissue; Table S2: Sequence alignment analysis table; Table S3: RNA-seq sequencing data statistics; Table S4: Differential gene expression between groups (Gene Set).
