# Supplementary material for: (-)-Gallocatechin Gallate Mitigates Metabolic Syndrome-Associated Diabetic Nephropathy in db/db Mice
Source: Foods. 2024 Jun 3;13(11):1755. doi: 10.3390/foods13111755 (PMC11171689; doi:10.3390/foods13111755)
Supplement: Supplementary file 1 [file foods-13-01755-s001.zip › foods-2983792-supplementary.pdf]

Supplement Table Captions

Table S1. Quality assessment of total RNA in kidney tissue.

Sample	Concentration (ng/μL)	Content (μg)	OD260/280	OD260/230	RQN
WT_1	1399.2	48.97	2.04	2.25	9.8
WT_2	1230.6	43.07	2.05	2.25	9.8
WT_3	1365a	47.78	2.04	2.26	9.8
WT_4	1474.3	51.6	2.06	2.25	9.8
WG_1	1237.3	43.31	2.02	2.25	9.8
WG_2	1095.6	38.35	2.01	2.26	9.8
WG_3	1147.9	40.18	2.02	2.26	9.8
WG_4	1219	42.67	2.03	2.25	9.4
M_1	1363.9	47.74	2.03	2.27	10
M_2	1666	58.31	2.03	2.24	10
M_3	1569.4	54.93	2.04	2.25	8.7
M_4	1671.8	58.51	2.04	2.25	9.9
GCG_1	1397.5	48.91	2.03	2.27	10
GCG_2	1474.2	51.6	2.03	2.25	9.9
GCG_3	1598.6	55.95	2.04	2.25	10
GCG_4	1568.3	54.89	2.03	2.26	9.9

Table S2. Sequence alignment analysis table.

Sample	Total reads	Total mapped	Multiple mapped	Unique mapped
WT_1	43016164	41590283(96.69%)	4095720(9.52%)	37494563(87.16%)
WT_2	48120776	46702062(97.05%)	4443191(9.23%)	42258871(87.82%)
WT_3	70067890	67977257(97.02%)	6942223(9.91%)	61035034(87.11%)
WT_4	42988770	41632569(96.85%)	4235378(9.85%)	37397191(86.99%)
WG_1	43762402	42352699(96.78%)	3911713(8.94%)	38440986(87.84%)
WG_2	42332774	40929297(96.68%)	3910509(9.24%)	37018788(87.45%)
WG_3	45639540	44261952(96.98%)	3885917(8.51%)	40376035(88.47%)
WG_4	49817154	48309263(96.97%)	4159627(8.35%)	44149636(88.62%)
M_1	44963264	43471636(96.68%)	4610777(10.25%)	38860859(86.43%)
M_2	40828694	39479902(96.7%)	4242752(10.39%)	35237150(86.3%)
M_3	47447964	45861864(96.66%)	5092994(10.73%)	40768870(85.92%)
M_4	45185334	43793927(96.92%)	4967623(10.99%)	38826304(85.93%)
GCG_1	43482390	42118817(96.86%)	4354033(10.01%)	37764784(86.85%)
GCG_2	45146664	43791374(97.0%)	4527756(10.03%)	39263618(86.97%)
GCG_3	41043186	39677185(96.67%)	4374838(10.66%)	35302347(86.01%)
GCG_4	52328994	50806403(97.09%)	5503602(10.52%)	45302801(86.57%)

Table S3. RNA-seq sequencing data statistics.

Sample	Raw reads	Raw bases	Clean reads	Clean bases	Error rate(%)	Q20(%)	Q30(%)	GC content(%)
WT_1	43325142	6542096442	43016164	6395811705	0.0271	97.3	92.17	47.71
WT_2	48419976	7311416376	48120776	7129114713	0.0258	97.76	93.41	48.61
WT_3	70552882	10653485182	70067890	10403682278	0.0261	97.63	93.09	48.21
WT_4	43297932	6537987732	42988770	6369616181	0.0262	97.62	93.06	48.14
WG_1	44095034	6658350134	43762402	6490756825	0.0266	97.44	92.67	48.62
WG_2	42625480	6436447480	42332774	6283987288	0.0265	97.51	92.76	48.19
WG_3	45941638	6937187338	45639540	6761673536	0.026	97.66	93.21	49.22
WG_4	50222352	7583575152	49817154	7380933296	0.0263	97.53	92.92	49.45
M_1	45284664	6837984264	44963264	6641405957	0.0261	97.65	93.16	48.43
M_2	41126498	6210101198	40828694	6018422547	0.0262	97.59	93	48.35
M_3	47825208	7221606408	47447964	6982611426	0.0266	97.44	92.68	48.35
M_4	45492826	6869416726	45185334	6641326949	0.026	97.7	93.26	48.37
GCG_1	43882200	6626212200	43482390	6441000417	0.0265	97.49	92.74	48.63
GCG_2	45466358	6865420058	45146664	6645399509	0.0261	97.65	93.19	48.91
GCG_3	41389524	6249818124	41043186	6048128889	0.0268	97.38	92.53	48.29
GCG_4	52872856	7983801256	52328994	7681937503	0.026	97.65	93.21	48.84

Table S4. Differential gene expression between groups (Gene Set).

Gene name	M/WT			GCG/M		
	Log2FC	Pvalue	Regulate	Log2FC	Pvalue	Regulate
AA467197	2.887549575	3.51E-08	up	-1.163446005	0.004790211	down
Acod1	5.950155804	2.49E-11	up	-4.536010389	7.08E-13	down
Adam8	1.71065724	5.06E-06	up	-3.08460503	5.52E-10	down
Adamdec1	1.590741235	0.040600364	up	-1.789405489	0.041214975	down
Adamts13	3.074975507	7.55E-60	up	-1.101187	6.28E-18	down
Anxa8	1.782946339	1.45E-07	up	-1.638222306	4.67E-07	down
Areg	4.306919624	0.004159059	up	-3.442674084	0.017646148	down
Arg1	4.535108722	0.001947315	up	-3.669847971	0.009788368	down
Cbr2	2.153911127	8.74E-27	up	-1.445393289	2.46E-14	down
Ccl3	2.699103795	2.15E-06	up	-5.249244623	1.56E-06	down
Ccl8	1.574207673	0.008955644	up	-2.02812242	0.003577598	down
Ccr1	1.763727598	1.47E-05	up	-2.382537633	7.35E-08	down
Cd177	5.412554712	2.79E-06	up	-2.754841405	0.000198755	down
Cd200r4	2.582474559	0.02893293	up	-3.035132184	0.020187192	down
Cd300lf	2.714967893	4.62E-07	up	-2.286059398	7.76E-06	down
Cd44	1.374222572	1.75E-11	up	-1.123316086	1.60E-08	down
Cemip	1.047955843	0.011380905	up	-1.398336033	0.002591517	down
Chil3	2.264170108	8.03E-07	up	-1.073026049	0.005163668	down
Clec4d	3.347431628	0.000198397	up	-2.827714058	0.000580638	down
Clec4e	2.753411647	7.45E-05	up	-1.301694502	0.017301785	down
Col17a1	1.730049133	0.014540174	up	-2.801253595	0.002069275	down
Csf3	4.680528972	0.002433208	up	-4.537610415	0.003523371	down
Csf3r	2.154758924	1.98E-12	up	-1.419344025	1.82E-06	down
Cxcl1	2.690545996	9.96E-08	up	-1.065251422	0.005290452	down
Cxcl2	7.718733464	2.08E-13	up	-5.049890396	4.13E-17	down
Cxcl3	6.469638701	1.21E-08	up	-6.32593403	2.69E-08	down
Cxcl5	8.326380244	2.98E-15	up	-8.182292056	9.12E-15	down
Cxcr2	1.888638655	0.000819093	up	-1.751164517	0.001095262	down
Cyp27b1	4.422519453	0	up	-1.200174245	9.93E-159	down
Dio2	1.486628519	0.016374943	up	-1.735474341	0.005913562	down
Duox2	4.543420304	0.000369878	up	-2.715307304	0.009052229	down
Duoxa2	5.132454873	7.74E-05	up	-4.989011574	0.000130138	down
Fos	1.396664429	1.24E-11	up	-1.623420758	9.83E-15	down
Gabrp	3.018685722	3.51E-08	up	-4.981854056	6.88E-08	down
Gbp11	1.694980102	0.011090534	up	-1.728322244	0.013031176	down
Gm10300	1.445965857	0.00417432	up	-1.082109439	0.019957743	down
Gm12940	-2.060451963	1.40E-06	down	1.065377812	0.041805799	up
Gm13092	1.353133078	0.003162564	up	-1.562098505	0.00152459	down
Gm14029	3.234662143	0.032487946	up	-3.812142927	0.016873549	down
Gm14117	1.970670431	0.038492578	up	-3.205539009	0.015118976	down
Gm30054	-4.307982085	0.00347116	down	4.384169663	0.008183493	up
Gm31243	5.552695699	1.23E-06	up	-1.116812914	0.031722705	down
Gm31600	-2.216102943	1.37E-06	down	1.249040256	0.021934191	up
Gm37087	4.527507773	0.004043627	up	-4.383193046	0.005734212	down
Gm37320	-3.382874305	0.021746578	down	3.041003223	0.044214232	up
Gm37758	1.765234937	2.02E-10	up	-1.135768804	3.88E-06	down
Gm38246	3.352286071	3.87E-12	up	-1.179940549	0.00030871	down

Gm38431	-1.465665713	4.34E-10	down	1.469569149	0.003665901	up
Gm43549	-3.722876546	2.79E-12	down	1.48735971	0.045206181	up
Gm45220	-2.842682745	0.011143551	down	2.434125678	0.047039716	up
Gm48604	-1.282984744	0.033088363	down	1.428308761	0.017612493	up
Gm5784	-1.586808162	0.006300311	down	1.268052265	0.034095396	up
Gpr141	1.582253568	0.000963336	up	-1.393268068	0.002813253	down
Gpx2	4.160535223	0.000438287	up	-3.976060709	0.00113745	down
Gstm3	-2.36738036	0.01258606	down	1.407381288	0.036389378	up
Gzmb	2.811096046	9.43E-06	up	-2.28545617	0.000111102	down
Hcar2	3.875674009	6.05E-11	up	-2.888099364	1.67E-08	down
Hp	1.960907317	1.24E-10	up	-1.263361221	2.74E-07	down
Hsd17b1	-3.991738504	0.002785963	down	3.139713913	0.036212674	up
Igha	1.785145536	2.97E-203	up	-2.76539816	0	down
Ighe	4.498892975	0.000726587	up	-4.35405639	0.001138381	down
Ighg1	6.509720371	1.72E-197	up	-4.940744683	1.17E-263	down
Ighg2b	3.004698953	1.82E-198	up	-3.228042016	2.70E-194	down
Ighg2c	2.350087708	1.11E-112	up	-2.95920048	3.42E-148	down
Ighv1-19	1.142539331	0.001338041	up	-2.860584675	8.28E-09	down
Ighv1-26	1.098854352	0.010002826	up	-1.587549742	0.000779241	down
Ighv1-34	1.630998994	1.82E-06	up	-2.165664842	2.76E-08	down
Ighv1-39	2.155910034	0.001135419	up	-5.206516255	8.17E-06	down
Ighv14-2	2.060962206	0.009791023	up	-3.837028366	0.001472664	down
Ighv1-42	3.194422446	5.00E-06	up	-4.476327913	2.90E-06	down
Ighv14-3	3.129450719	7.51E-14	up	-2.54512438	4.84E-11	down
Ighv14-4	4.346499443	0.000817076	up	-4.923839196	0.000213292	down
Ighv1-49	3.938343013	0.011169867	up	-3.794330955	0.015069545	down
Ighv1-5	1.531721575	0.026525607	up	-3.938350658	0.000967214	down
Ighv1-50	1.286331075	0.003807107	up	-3.959561703	3.47E-07	down
Ighv1-52	6.193636084	1.93E-09	up	-6.758004434	3.31E-10	down
Ighv1-55	1.173886942	1.68E-05	up	-3.486582454	2.66E-18	down
Ighv1-62-2	3.135906655	0.030573982	up	-3.609863557	0.013918744	down
Ighv1-64	1.425166663	0.000573899	up	-2.354811674	2.16E-06	down
Ighv1-66	1.77247902	0.000134336	up	-3.104563372	1.04E-06	down
Ighv1-7	2.810304671	2.16E-10	up	-4.258228244	1.75E-11	down
Ighv1-72	2.073870756	1.10E-11	up	-3.667076922	3.87E-17	down
Ighv1-75	1.830361767	0.001630146	up	-6.420655476	1.60E-08	down
Ighv1-78	2.347329972	0.000129711	up	-6.309656702	2.57E-08	down
Ighv1-80	1.054981284	0.030780868	up	-5.633667821	5.47E-07	down
Ighv1-82	1.798681057	7.82E-08	up	-2.996818932	1.27E-13	down
Ighv2-2	3.894482101	2.78E-10	up	-5.159848109	1.65E-08	down
Ighv2-3	3.210194038	0.003609044	up	-4.400729846	0.00053577	down
Ighv2-5	3.993986075	2.56E-08	up	-4.074334264	9.14E-08	down
Ighv2-6	4.6588803	0.000461367	up	-2.409871699	0.027138248	down
Ighv3-6	1.89197546	0.005994302	up	-2.919105711	0.000739588	down
Ighv4-1	4.784885319	1.46E-20	up	-6.35109717	2.66E-13	down
Ighv5-12	2.877614152	0.010819728	up	-2.344466223	0.031346345	down
Ighv5-4	2.25739747	1.36E-05	up	-1.944114282	6.05E-05	down
Ighv5-6	4.56452253	7.70E-10	up	-5.71126224	9.16E-08	down
Ighv5-9	1.774516088	0.000261923	up	-4.643930074	8.18E-07	down
Ighv5-9-1	5.147185997	1.45E-05	up	-3.344728689	0.000698133	down
Ighv7-4	3.353593035	7.47E-05	up	-5.188319693	8.63E-06	down

Ighv8-12	2.165778496	0.000678385	up	-4.778357895	2.28E-05	down
Ighv8-8	3.263834905	1.47E-07	up	-4.818131062	3.58E-07	down
Ighv8-9	2.689904638	0.000108705	up	-6.123632648	1.27E-07	down
Ighv9-2	4.719346043	0.000520196	up	-4.575388065	0.00081038	down
Ighv9-3	4.551647715	3.32E-24	up	-5.191467905	1.30E-20	down
Igkc	2.368531019	5.46E-155	up	-2.931543553	1.49E-270	down
Igkv10-94	3.015777428	2.18E-09	up	-5.771551126	6.23E-08	down
Igkv10-96	1.835889293	2.84E-15	up	-2.42436109	3.50E-18	down
Igkv1-122	5.601440528	5.77E-10	up	-7.436802031	5.09E-12	down
Igkv1-135	1.615549635	1.11E-05	up	-3.460821808	2.80E-11	down
Igkv12-41	3.655751832	0.032109284	up	-3.511542246	0.040832459	down
Igkv12-44	2.252716888	2.58E-05	up	-2.641964771	5.24E-06	down
Igkv12-46	1.622987613	0.002912619	up	-1.07695451	0.028065261	down
Igkv12-98	6.428501304	6.64E-09	up	-5.578793257	3.41E-07	down
Igkv14-100	3.671659802	0.002580584	up	-4.215350926	0.000925931	down
Igkv14-111	2.112343712	4.02E-14	up	-2.386318797	4.93E-16	down
Igkv14-126	4.214268902	0.001142723	up	-3.361965345	0.007669426	down
Igkv15-103	2.425987937	1.99E-11	up	-4.684593375	9.31E-18	down
Igkv17-121	2.545232777	0.009190894	up	-3.133095357	0.004723519	down
Igkv17-127	5.88493815	3.51E-07	up	-5.740442338	7.12E-07	down
Igkv1-99	2.819526571	0.004497221	up	-5.056319064	7.31E-05	down
Igkv19-93	1.920228379	9.65E-11	up	-4.488634809	8.32E-21	down
Igkv2-137	4.279579817	7.67E-26	up	-6.197698413	1.87E-16	down
Igkv3-10	4.123156601	7.37E-17	up	-5.646647937	5.35E-13	down
Igkv3-2	1.245149884	3.44E-16	up	-2.628396363	2.20E-38	down
Igkv3-4	2.253189929	2.90E-31	up	-3.784096832	2.47E-55	down
Igkv3-5	2.456523359	1.24E-10	up	-4.972937482	2.75E-12	down
Igkv3-7	4.573536136	3.09E-131	up	-5.113688088	3.76E-21	down
Igkv4-53	3.33478681	2.90E-08	up	-4.199881337	3.87E-08	down
Igkv4-59	2.246402927	1.04E-10	up	-3.815645462	8.31E-16	down
Igkv4-61	1.361964023	3.24E-07	up	-3.091937486	1.25E-15	down
Igkv4-68	1.177371922	0.033249324	up	-1.41197566	0.017222254	down
Igkv4-74	4.650574236	1.03E-15	up	-3.882447095	2.82E-15	down
Igkv4-79	5.281014353	3.84E-05	up	-4.414823778	0.000491809	down
Igkv4-80	4.837332936	3.03E-17	up	-4.8639492	1.14E-15	down
Igkv4-86	3.066462916	0.046518178	up	-3.643425114	0.025670376	down
Igkv5-43	1.246335164	0.000152989	up	-3.98320764	2.77E-11	down
Igkv5-48	4.265613501	3.19E-06	up	-4.064559728	1.00E-05	down
Igkv6-17	2.359242429	1.30E-78	up	-3.643685552	2.37E-106	down
Igkv6-20	3.37654534	1.60E-07	up	-4.338060342	2.15E-07	down
Igkv6-23	1.534859237	1.11E-08	up	-3.177916157	6.61E-18	down
Igkv6-25	3.980361249	5.12E-33	up	-4.460551857	3.86E-31	down
Igkv6-32	1.638112046	0.003971463	up	-1.871696818	0.003028009	down
Igkv8-21	3.725185546	1.10E-14	up	-2.908336242	3.60E-12	down
Igkv8-28	2.11519106	0.013755705	up	-5.173916891	4.38E-05	down
Igkv8-30	1.32764094	0.001051926	up	-6.19610201	1.35E-08	down
Igkv9-120	1.647930975	0.007533117	up	-4.447570312	0.000118081	down
Igkv9-124	2.49821675	0.001425074	up	-3.132446062	0.000795608	down
Iglc2	1.041068969	0.00882508	up	-1.943923553	1.74E-05	down
Iglv1	2.175864665	1.98E-45	up	-3.85232515	1.26E-69	down
Il1a	3.126319908	0.017559311	up	-3.666154095	0.007995025	down

Il1b	1.917435652	1.37E-13	up	-3.637638617	8.52E-22	down
Il1r2	2.884294318	6.58E-05	up	-3.116412626	8.67E-05	down
Il1rn	4.411057307	2.57E-14	up	-4.403736892	3.19E-13	down
Il36g	4.039473373	5.94E-05	up	-3.823604911	0.000169878	down
Jchain	1.437422623	4.39E-71	up	-2.645426728	1.43E-127	down
Krt14	3.077265943	3.63E-11	up	-4.637013174	3.49E-12	down
Krt15	1.811267829	3.59E-17	up	-1.251977086	5.16E-10	down
Krt19	1.340425014	1.73E-18	up	-1.071515537	7.18E-13	down
Krt5	3.060404045	1.19E-58	up	-2.956869846	4.87E-52	down
Lcn2	3.131787526	1.70E-112	up	-2.828689194	1.39E-101	down
Lif	2.08188643	0.000144682	up	-1.175933551	0.013519349	down
Lilrb4a	1.074120932	0.000545826	up	-1.427695919	2.78E-05	down
Lilrb4b	1.244385975	5.97E-05	up	-1.196485442	0.000110049	down
Lrat	2.661339168	1.36E-05	up	-1.147325712	0.049912709	down
Ltbp2	1.443790314	2.79E-05	up	-1.686411653	2.31E-06	down
Ltf	5.439293396	1.04E-51	up	-4.204088155	1.00E-54	down
Ly6d	2.229485475	2.43E-23	up	-1.315364508	1.74E-11	down
Lyve1	1.062166428	0.031445741	up	-1.043722432	0.033184673	down
Mcpt1	5.074080158	8.88E-05	up	-4.930171739	0.000148698	down
Mcpt2	5.635528335	9.22E-06	up	-3.02806537	0.00287531	down
Mefv	2.084742504	0.019209182	up	-2.764749399	0.006949275	down
Meltf	1.502369097	0.043424597	up	-1.547911591	0.040219621	down
Mmp3	1.103302525	0.010489716	up	-3.80424933	8.55E-09	down
Mmp7	4.762635605	6.97E-20	up	-4.751256937	2.57E-18	down
Mmp9	1.321659593	0.001079152	up	-2.093779492	7.54E-06	down
Ms4a6d	1.059537012	0.027643103	up	-1.03919187	0.027366739	down
Muc5b	5.316435005	2.62E-05	up	-4.45104419	0.000375163	down
Mzb1	1.559806483	0.000842622	up	-2.025178376	0.000152795	down
Nkx6-2	3.82394197	0.00309913	up	-2.626505051	0.015091266	down
Nlrp3	1.263417203	0.001061845	up	-1.790994628	8.38E-05	down
Osm	4.091212276	0.00059224	up	-5.324080776	1.67E-05	down
Padi1	3.567463564	0.000604899	up	-2.993313827	0.001453333	down
Pakap	-3.596050193	0.010326979	down	4.110044446	0.001630284	up
Pdcd1	1.490719429	0.030517744	up	-2.985847915	0.002036248	down
Peg10	1.433320624	2.01E-05	up	-1.325436685	0.000157735	down
Pfn3	-1.649274683	0.009251777	down	1.594878983	0.008245076	up
Pira2	1.855009337	0.013485071	up	-2.238734679	0.02298811	down
Plin1	-1.198773421	0.020894903	down	1.060825807	0.049598058	up
Pou2af1	1.487582781	0.002230835	up	-2.924686091	1.36E-06	down
Pram1	2.591574978	0.000163851	up	-1.18736898	0.036354688	down
Prss22	5.061538328	4.88E-08	up	-2.60273582	2.45E-06	down
Prss27	3.250829859	0.003358749	up	-2.026484568	0.024931332	down
Ptgs2	2.694773671	1.09E-09	up	-1.320919997	0.000170038	down
Reg3g	5.878711833	1.01E-17	up	-7.742966623	1.36E-13	down
Retnlg	3.155456516	1.56E-05	up	-2.579858136	7.94E-05	down
Rprm	1.588598094	0.004262212	up	-1.097296525	0.044364155	down
S100a8	3.00032634	3.61E-08	up	-3.465048878	3.96E-11	down
S100a9	3.398713993	2.22E-37	up	-3.400198307	4.73E-36	down
Saa2	3.616129078	0.034700642	up	-3.4727307	0.043885271	down
Selp	2.835880216	3.33E-09	up	-2.104344354	6.65E-06	down
Serpina3f	3.048149764	1.42E-11	up	-1.95583725	1.63E-07	down

Serpina3g	1.954228331	3.02E-22	up	-1.28540785	2.09E-10	down
Serpina3h	2.92822099	0.003095208	up	-1.659929954	0.020044838	down
Serpina3n	2.448734879	3.34E-25	up	-2.53798762	1.71E-25	down
Serpinb5	1.55675573	0.03834801	up	-1.611086676	0.036293322	down
Sftpd	4.022863696	9.40E-05	up	-3.420363836	0.000275538	down
Siglec1	1.643291601	0.000328039	up	-1.427840101	0.00136197	down
Slc39a4	2.199892938	3.25E-08	up	-1.905096707	3.80E-07	down
Slc7a11	2.167298036	5.99E-05	up	-2.307840477	2.85E-05	down
Slfn4	2.113846202	0.002418983	up	-5.158518138	1.05E-05	down
Slpi	5.816383743	5.96E-42	up	-4.304246671	1.16E-48	down
Socs3	1.859844122	9.14E-20	up	-1.607324249	4.83E-18	down
Sprr2a1	1.915284958	0.00049694	up	-1.165275393	0.006309424	down
Sprr2a2	2.477733371	1.03E-94	up	-2.110678666	5.74E-79	down
Sprr2a3	2.40872909	1.67E-05	up	-1.215545681	0.008569767	down
Sprr2d	6.146779949	1.02E-07	up	-6.002730298	2.15E-07	down
Sprr2e	3.785533425	0.017885466	up	-3.641690816	0.023525629	down
Sprr2f	7.557427948	7.43E-13	up	-7.413623389	2.06E-12	down
Sprr2g	3.207237004	0.003087662	up	-4.336574614	0.00060393	down
Tmprss11g	6.391588071	2.65E-08	up	-6.248136614	5.77E-08	down
Tnfrsf9	2.347991713	0.012082374	up	-1.747919149	0.048991146	down
Tnfsf8	1.825189176	0.035633177	up	-2.260630694	0.016385869	down
Tnip3	1.348352849	0.029553864	up	-3.046747314	0.000388454	down
Trem1	6.133079408	2.74E-08	up	-2.119153969	3.43E-05	down
Ttyh1	-1.610596946	0.00109918	down	1.164564744	0.028786366	up
Xlr3a	-4.565223649	3.30E-15	down	2.173570381	0.005383351	up
Xlr3b	-2.405612241	1.94E-20	down	1.450088085	1.51E-05	up
Zan	1.428852462	0.009313214	up	-1.343203937	0.012654305	down
